# A message from the President of ANAMT

**DOI:** 10.5327/Z167944352019v17n4EDT

**Published:** 2019-12-01

**Authors:** Rosylane Nascimento das Mercês Rocha

**Affiliations:** President of National Association of Occupational Medicine. USA USA

I am extremely glad for this first contact with the readership of our Revista Brasileira de Medicina do Trabalho as the new president of the National Association of Occupational Medicine (ANAMT).

I would like to congratulate and thank the Editorial Board for having made our journal a high-quality publication. Congratulations to you all, and thank you so much for your work and the results attained until now.

We are making some changes in the journal management. Aware of the many challenges we have to overcome, we intend to meet them by raising even more the quality of our journal. Within this context, the occupational physician, Dr. Andrea Franco Amoras Magalhães, MD PhD, was appointed as the new editor-in-chief to meet the requirement for inclusion in Scientific Electronic Library Online (SciELO) and achieve a higher ranking in the Brazilian Agency for Support and Evaluation of Graduate Education (CAPES) database Qualis.

In addition, we invited international editors to join the new Editorial Board, as well as occupational physicians affiliated with higher education institutions-all of them committed to high-quality scientific research. Thus we intend to actualize our commitment to extend ANAMT’s reach to all regions in Brazil.

Serious scientific production is an indispensable requirement to make occupational medicine stronger in Brazil and abroad. For this purpose we created a special committee to encourage and support occupational physicians interested in publishing their studies as scientific articles to choose our journal.

Stay on with the news! A lot more to come soon!

